# How Do Smut Fungi Use Plant Signals to Spatiotemporally Orientate on and *In Planta*?

**DOI:** 10.3390/jof7020107

**Published:** 2021-02-02

**Authors:** Karina van der Linde, Vera Göhre

**Affiliations:** 1 Department of Cell Biology and Plant Biochemistry, University of Regensburg, 93053 Regensburg, Germany; 2 Institute for Microbiology, Cluster of Excellence on Plant Sciences, Heinrich-Heine University, 40225 Düsseldorf, Germany

**Keywords:** *Ustilago*, *Microbotryum*, *Sporisorium*, *Thecaphora*, nutrition, meristem, growth, development

## Abstract

Smut fungi represent a large group of biotrophic plant pathogens that cause extensive yield loss and are also model organisms for studying plant–pathogen interactions. In recent years, they have become biotechnological tools. After initial penetration of the plant epidermis, smut fungi grow intra—and intercellularly without disrupting the plant-plasma membrane. Following the colonialization step, teliospores are formed and later released. While some smuts only invade the tissues around the initial penetration site, others colonize in multiple plant organs resulting in spore formation distal from the original infection site. The intimate contact zone between fungal hyphae and the host is termed the biotrophic interaction zone and enables exchange of signals and nutrient uptake. Obviously, all steps of on and *in planta* growth require fine sensing of host conditions as well as reprogramming of the host by the smut fungus. In this review, we highlight selected examples of smut fungal colonization styles, directional growth *in planta*, induction of spore formation, and the signals required, pointing to excellent reviews for details, to draw attention to some of the open questions in this important research field.

## 1. Introduction

**Figure 1:**
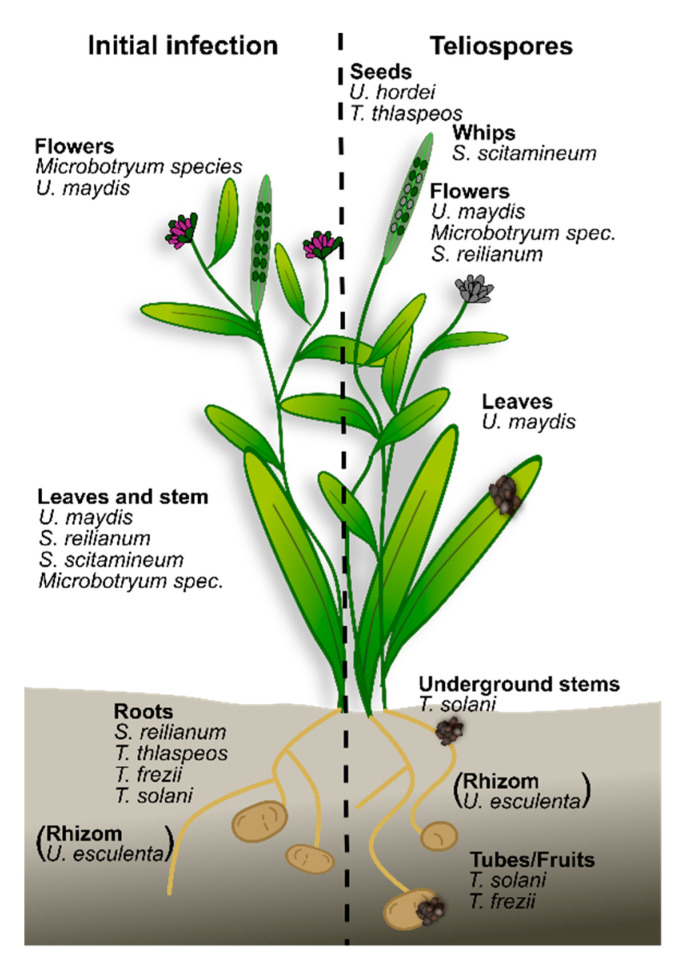
Overview of the different smuts discussed in this review. For each smut, the initial penetration sites and the site of teliospore formation is indicated. *T. thlaspeos* and *S. scitamineum* overwinter inside the host. *U. esculenta* is transmitted via the rhizome.

More than 1000 teliospore-forming fungi, which cause plant smut diseases, have been identified so far. The term smut stems from the German term “schmutzig” (English: dirty) and regards the appearance of infected plant material, which is caused by a massive amount of black, melanized teliospores. The vast majority of smut fungi infect angiosperms, and while most prefer annuals, some infect perennial plants [1]. Commonly, smuts are biotrophic pathogens exhibiting a narrow host range. Besides conventional phylogenetic classification, the smuts can be separated into three groups: (1) smut fungi that infect locally and form teliospores at the original penetration site; (2) smut fungi that infect systemically and colonize several tissues of their host, with teliospore production distant from the infection site; (3) smut fungi that can infect both locally and systemically (Figure 1). Furthermore, the life cycle of the host plant, e.g., annual vs. perennial, and the infected plant tissues, e.g., aerial vs. below-ground, are valuable points of comparison, since they differ in plant signals providing cues for the fungus (Figure 1). In this review, we compare the different infection strategies used by smut fungi and summarize the underlying signals that enable spatiotemporal orientation of the fungus on and in the plant. 

## 2. Smut Fungi Differ in Their Infection Strategies

To colonize their host plant, all smut fungi face the initial challenge of penetrating the plant tissue, and subsequently need to proliferate to ultimately sporulate for fungal propagation (Figure 1). The life cycle is best characterized in the model smut fungus *U. maydis* [2]. Teliospores landing on a plant surface germinate, giving rise to haploid sporidia that can proliferate asexually. Pathogenic development of *U. maydis* starts with mating of sporidia, which leads to the formation of an infectious, dikaryotic filament [3]. This filament is arrested in the G2 phase of the cell cycle [4], and strong polar tip growth is supported by insertion of retraction septa at the basal pole while maintaining cytoplasmic continuity [5]. Both the cell cycle arrest and septation are required for the formation of appressoria for penetration [6]. These appressoria are non-melanized and do not build up turgor pressure, but rather rely on plant-cell-wall-degrading enzymes [7] that are induced by plant surface cues such as the hydrophobic surface and cutin monomers [8]. Successful penetration of the cuticle leads to re-activation of the cell cycle without nuclear fusion and subsequent local biotrophic proliferation of the dikaryotic filament. During the biotrophic phase, hyphae grow inter—and intracellularly in the plant tissues. Several reviews describe effector translocation and its activity in suppressing the host-defense responses during the biotrophic phase [9,10,11]. Induction of plant tumors depends on specific effectors, such as See1 (seedling efficient effector 1) [12], but is independent of the subsequent sporulation that is controlled by the transcription factor ROS1 (regulator of sporogenesis 1) [13]. Ultimately, karyogamy and sporulation occur in the tissue adjacent to the penetration site as early as within 12 days [14]. This makes *U. maydis* a representative of the locally infecting smut fungi. 

Organ-specific, local infection also occurs when *Microbotryum* species infect flowers of the white campion, *Silene latifolia* (Figure 1). Upon invasion of male flowers, the fungus sporulates in pollen sacs. During the infection of female flowers, these are turned into a male morphology to promote fungal proliferation [15,16]. In addition, *Microbotryum* is also able to grow systemically when it infects seedlings [17]. Interestingly, the germination pattern of teliospores differs between seedlings and flowers. Germination of seedlings leads to immediate mating and penetration of the host plant in the filamentous form, while germination in flowers initially permits budding of sporidia and yeast-like growth after germination. Different nutrient conditions—limiting on seedlings, rich in flowers—might explain this difference [17]. Hence, the timing and location of infection can influence the life cycle of the fungus, whether they proceed quickly to sporulation or spread systemically in the host plant in an endophytic manner.

Closely related to *U. maydis* are the two grass smut fungi *S. reilianum* and *U. hordei*. *S. reilianum* causes head smut in maize (*Zea mays*) and sorghum (*Sorghum bicolor*), *U. hordei* causes covered smut in barley (*Hordeum vulgare*) and oat (*Avena sativa*). Their life cycles closely resemble *U. maydis* with a dimorphic switch during infection and biotrophic infection of the respective host plant. However, *in planta,* there are interesting differences in comparison to *U. maydis* (Figure 1): Both fungi infect their host plant systemically instead of locally; they sporulate only in the reproductive organs; they do not induce plant tumors. For *S. reilianum*, two infection routes are known. First, it can infect the roots to proliferate endophytically until it reaches the stem [18,19]. At this stage, fungal proliferation can be restricted by the plant immune system in resistant maize cultivars [20]. Second, similar to *U. maydis*, *S. reilianum* can directly infect aerial tissues. This causes changes in the anatomy of maize ears and tassels. As a result of infection-induced alterations in floral organ identity and meristem determinacy, the cobs are turned into leafy cobs [21]. In addition, the fungus triggers suppression of apical dominance, which leads to a higher number of female inflorescences (ears) in maize [21] and increased tillering of sorghum [22]. Notably, both *U. maydis* and *S. reilianum* alter host-plant morphology in maize: *U. maydis* induces tumor formation, *S. reilianum* causes phyllody. This might suggest that maize as a host plant is prone to morphological changes. In contrast to the maize pathogens *U. maydis* and *S. reilianum*, *U. hordei* infects barley and oat. Colonization occurs at the seedling stage, and similar to *U. maydis* and *S. reilianum*, it requires mating to form infectious filaments and to penetrate via appressoria. After penetration, the dikaryotic filaments grow intercellularly towards the vascular tissue for the first few days. Reaching this transport tissue, hyphae continue to grow, but also form feeding structures resembling haustoria when the fungus invades a host cell [23]. Such structures have not been described for *U. maydis* or *S. reilianum* to date. Subsequently, *U. hordei* grows in or below the shoot meristem during the biotrophic phase, and finally sporulates in the spikelets [24] (Figure 1). Notably, *U. hordei* does not seem to manipulate the meristem and does not cause any morphological changes until it replaces seeds with teliospores during sporulation. 

While *U. maydis*, *S. reilianum*, and *U. hordei* infect annual crop plants, *S. scitamineum*, which has been threatening agricultural production worldwide for the last century, is a pathogen of the perennial sugarcane (*Saccharum officinarum*) (reviewed in [25]). Similar to *U. maydis* and *S. reilianum*, it alters plant morphology by inducing a whip-like sorus consisting of plant and fungal cells for sporulation [26] (Figure 1). Fungal teliospores spread from the whips and germinate on the plant surface or in the soil, giving rise to sporidia. After mating, the dikaryotic filament penetrates the young bud via appressoria, and hyphae proliferate systemically colonizing the apical meristems [27]. Notably, infected buds can either show symptoms by inducing whips with the fungus proceeding to sporulation, or they remain asymptomatic, but filled with hyphae that are dormant in the plant until the following season (reviewed in [25]). In comparison to the annual smut fungi, the decision between sporulation and vegetative growth is an important open research question for perennial smut fungi. 

Similar to *S. scitamineum*, *U. esculenta* also infects a perennial host plant, wild rice (*Zizania latifolia),* and causes swollen stems, which are consumed as a vegetable in Asian countries [28]. A unique feature of this fungus is its spreading via the plant rhizome [29]. Upon systemic colonization, *U. esculenta* suppresses flowering in wild rice, induces stem galls, and proliferates together with the host rhizome in its vegetative form [29]. In this state, it has been maintained by Chinese farmers for more than 2000 years, but only recently do molecular studies elucidate the infection mechanisms, which are supported by sequencing of the fungal genome [30]. Two more examples for smut fungi propagating via underground tissues are *Thecaphora solani* [31] and *T. frezii* [32]. These dicot smut fungi infect potato (*Solanum tuberosum*) and peanut (*Arachis hypogaea*), respectively. *T. solani* induces galls on underground stems, stolons and tubers [33], *T. frezii* replaces the peanut as a seed by teliospores [34] (Figure 1). In contrast to *U. esculenta*, which propagates underground in the vegetative, hyphal form, both fungi sporulate in the below-ground reproductive organs (tubers and gynophore) [31,32], and teliospores remain stable in the ground for several years [35]. A third example for a perennial pathogen is the Brassicaceae smut fungus *T. thlaspeos*. It infects *Arabis* species, but also other Brassicaceae [36], hence it has a slightly broader host range than the grass smut fungi. Interestingly, in the *Thecaphora* species, germination differs from the grass smut fungi since filaments emerge from the teliospores that proliferate [31,37,38]. Hence, there is not a morphological switch from a saprophytic, yeast-like growth phase to an infectious filament. Nevertheless, *T. thlaspeos* has retained the mating genes, and filamentous cultures of compatible mating types are able to mate resulting again in a filament [37]. In contrast to *S. scitamineum*, which has not been reported to infect roots so far, and *U. esculenta,* which proliferates via the rhizome and concurrently suppresses flowering, *T. thlaspeos* can infect both roots and aerial tissues (Figure 1). Typical for smut fungi, it sporulates in the siliques by replacing seeds with teliospores, but it can also overwinter in the root tissue together with the winter-hard host species for several seasons to sporulate in accord with flowering of the host plant [37]. Macroscopically, no symptoms are detectable, and *T. thlaspeos* is able to moderate its virulence during the biotrophic growth along the vasculature, enabling the long-lived infection of its perennial host [39]. Molecular studies in the model plant A. thaliana and a newly established transformation protocol for the fungus will in the future greatly contribute to understanding the underlying signals, which the fungus perceives to switch between biotrophic growth and sporulation [40,41].

## 3. Signals Directing Growth and Development

### 3.1. Signals on Planta

To orient inside the plant and coordinate the lifecycle with plant development, smut fungi need to perceive plant signals. This already starts prior to initial contact with the host plant with the decision of teliospores to germinate or remain dormant. In many smut fungi, teliospores rapidly germinate, yet some species require germination signals. For example, *T. thlaspeos* teliospores remain dormant under “standard” smut germination conditions. Co-incubation with pre-germinated plant seeds induces germination reaching rates up to 76%. The unknown plant dormancy breaking signal is heat stable and not host-specific [37]. Teliospores of *Urocystis agropyri*, which causes flag smut in wheat, start germinating only after sowing of wheat. As described for *T. thlaspeos,* germination is induced by host and non-host tissues. More detailed analysis revealed that volatiles from various plant resources strongly induce germination. These results led to the hypothesis that ethylene might be the main driver of germination [42], but *T. thlaspeos* teliospores do not respond to ethylene [43].

In *U. maydis,* cell fusion and filamentous growth can be induced by pheromone signaling in the absence of host plants, but appressorium formation requires perception of plant signals. The hydrophobic surface of the leaves is the main cue for appressoria development. Notably, 16-hydroxy hexadecanoid acid, which is a cutin monomer, strongly enhances appressorium formation efficiency [44]. The transmembrane osmo-sensor Sho1 and the transmembrane mucin Msb2 mediate the sensing of the hydrophobic surface and are required for appressorium formation [45]. The MAP-kinase Kpp2 acts downstream of Sho1 and Msb2 in filament and appressorium formation on a hydrophobic surface [44]. In addition MAP-kinase Kpp6 is essential for appressorium function [46]. These genes are highly conserved in smut fungi, but interestingly *U. esculenta*, which can proliferate together with the host rhizome, lacks a sho1 orthologue [30,47].

Upon successful penetration, smut fungi have to face two major challenges: (1) overcoming the plant defenses and (2) acquiring nutrition from the host. Interaction of smuts with the plant-defense system, especially the importance of secreted effector proteins, has been summarized in other reviews (see [10,48]). Some of these effectors are transcriptionally induced early on by plant surface cues [8]. Similarly, expression of fungal nitrogen and sugar transporters is induced by hydrophobicity and 16-hydroxy hexadecanoic acid indicating that the fungus prepares for biotrophic interaction and nutrition uptake already before penetration [8,14]. 

### 3.2. Nutrition as Signals for Directional Growth

The literature describes directional growth towards the vasculature after initial penetration of the plant for several smut fungi. Plants use the vasculature to transport amino acids, RNAs, organic acids, vitamins, and soluble carbohydrates in the phloem, and minerals are transported by bulk flow with water in the xylem. Bundle sheath cells surround the vasculature and mediate loading and unloading of nutrients. Hence, smut fungi growing close to vascular tissues might sense the nutrient gradients to locate themselves close to accessible nutrients of the host plant. There, plasma membrane localized transporters mediate the nutrient uptake from the plant apoplast. 

**Figure 2:**
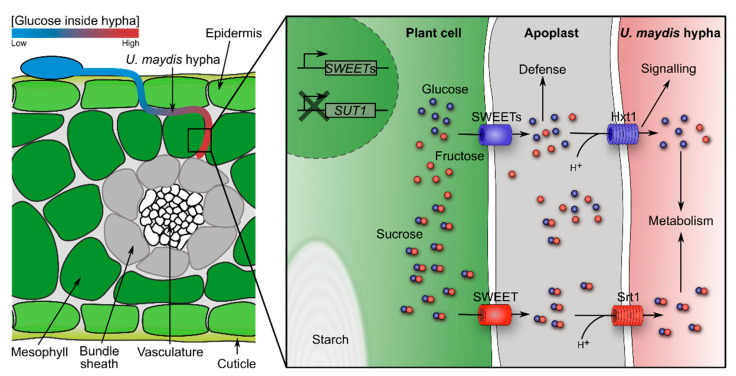
Schematic overview of sugar flux in *U. maydis*—infected leaf tissue. A *U. maydis* hypha grows between epidermis and mesophyll cells towards the bundle sheath and vasculature. Glucose accumulates towards the hypha tip region. Upon infection, *U. maydis* induces expression of *SWEET*s, which leak hexoses and sucrose into the apoplast. These sugars are taken up by the fungus via Srt1 and Hxt1. *U. maydis* blocks expression of *SUT1* to prevent phloem loading, subsequently starch accumulates.

During *U. maydis* infection of seedling, leaf sugars and amino acids accumulate in the infected tissue turning them into strong sinks [49,50,51,52]. Based on gene expression profiles and mutant analyses, it was proposed that nitrogen uptake in the form of amino acids is dependent on activity of proteases, which are secreted by *U. maydis*, and subsequent uptake of peptides is mediated by fungal oligopeptide transporters. Ammonium uptake is postulated to be facilitated by the ammonium transporters Ump1 and Ump2, since double mutants are severely compromised in virulence. By contrast, urea uptake does not seem to be required during infection of seedling leaves [14]. Sugars are energy and carbon sources for the fungus. Two out of 19 sugar transporters (Srt1 and Hxt1) are essential for infection (Figure 2). Srt1 is the fungal sucrose transporter that provides sucrose as a carbon and energy source for the fungus. It shows high similarity to uncharacterized transporters from other smuts suggesting that sucrose is a major carbon source for many smut fungi [27,53,54]. The hexose transporter *hxt1* is constitutively expressed in *U. maydis* and shows similarities to the *S. cerevisiae* glucose receptors Snf3p and Rgt2p. Complementation of the SG200Δ*hxt1* mutant, which is severely impaired in pathogenicity, with other high affinity hexose transporters unexpectedly did not restore virulence. Furthermore, *U. maydis* strains harboring point mutations in *hxt1,* which induce constitutive active glucose signaling, are blocked immediately after plant penetration. This indicates that Hxt1 might have a sensor function besides its transport activity (Figure 2). Interestingly, expression of fungal effectors is disturbed in these *hxt1*-mutant strains further supporting that *U. maydis* responds to glucose levels by adjusting its virulence [54]. *In planta* microscopic imaging of *U. maydis* strains expressing a cytoplasmic glucose FRET (Förster resonance energy transfer) sensor revealed a glucose gradient within the growing hyphae, where highest glucose concentration was detected in the tip (Figure 2). This fungal sugar gradient was abolished in infected leaves which did not contain soluble sugars after dark treatment [52]. Different scenarios can explain the fungal glucose gradient: (1) sugar metabolism could differ within the hyphae, or (2) the sugar import machinery could be tip polarized, or (3) sugar transporter turnover is accelerated at the tip, or (4) more glucose is available for uptake at the tip. The latter would correspond well with the absence of the gradient in dark-treated leaves. Taken together these data suggest that sugar sensing/signaling in *U. maydis* is strongly linked to biotrophic interaction and directional growth.

Sugar gradients might not only direct growth within an organ towards the vasculature but could also direct growth of smut fungi inside the entire plant. Plant organs can generally be discriminated into sink and source tissues. Sugars are produced in source tissues, e.g., leaves, and from there are distributed via the vasculature into non-photosynthetic active, consuming tissue. These sink tissues possess a hierarchy in terms of sugar distribution: seeds > fleshy fruit parts, underground storage organs > shoot apices and newly developing leaves > cambium > roots (for review see [55]). Many of the here mentioned smuts seem to follow this hierarchy during systemic growth by directing their growth to the seeds for sporulation. However, sugar distribution in the plant is a dynamic process, which is influenced by the supply and demand. *U. maydis* modifies expression of various sugar transporters in an organ-specific way. In seedling leaves, soluble sugars and starch accumulate. Especially pathogen-induced hypertrophic mesophyll cells show large amounts of starch granules, which are normally only observed in bundle sheath cells [49,50,51,52]. In concordance, expression of maize *SUT1* (*SUCROSE TRANSPORTER 1*), which is involved in leaf sugar efflux by phloem loading, is reduced. By contrast, three maize *SWEET* transporters are upregulated after seedling infection (Figure 2) [52,56]. These carriers are most probably responsible for leakage of hexoses and sucrose into the apoplast [52]. In addition, induction of genes involved in light reaction, Calvin cycle, photorespiration, tetrapyrrole synthesis, as well as sucrose and starch synthesis, does not occur in infected seedling leaves. Ultimately, during infection, *U. maydis*’ demand for nutrition blocks the transition of seedling leaves from sink to source [49,52]. In tassels, which are a strong sink tissue in plants, hexose concentrations are increased while sucrose concentration is per se higher compared to seedling leaves. Expression of *SUT1* is not altered and only one *SWEET* is transcriptionally upregulated [52,56]. *U. maydis*’ high demand for nutrition even outcompetes the strongest maize sink, which is the ear with developing seeds [52]. One may hypothesize that *U. maydis*’ ability to create this nutrition-rich niche inside the plant is associated with its “local life style” *in planta*. Yet, it is unknown which signals from *U. maydis* are required for changing the sink-source transition and relationship in the maize plant.

Obviously, plant-produced sugars are a central energy source for pathogens and might direct growth inside the host, but these sugars also play a pivotal role in plant defenses. Two factors contribute to SWEET immunity: on the one hand, plants defend themselves by starvation of the intruders via effective sugar retrieval mechanisms from the apoplast; on the other hand, sugars play a role in plant defense signaling (for review see [57,58]). Thus, smuts have to balance their hunger for nutrition against the defense response they cause and keeping this balance most probably requires sensing of carbohydrates by the pathogen.

### 3.3. Signals Involved in Morphological Changes of the Host

Several smuts induce plant morphological changes visible to the eye before spore release. Colonialization of tissues by *U. maydis* results in tumor formation, whereas *S. reilianum* causes phyllody, and *S. scitamineum* induces whips, for example, [59,60,61]. At least for U. maydis, these modifications are proposed to allow easier excess of nutrition for the fungus as mentioned above [50,52].

**Figure 3:**
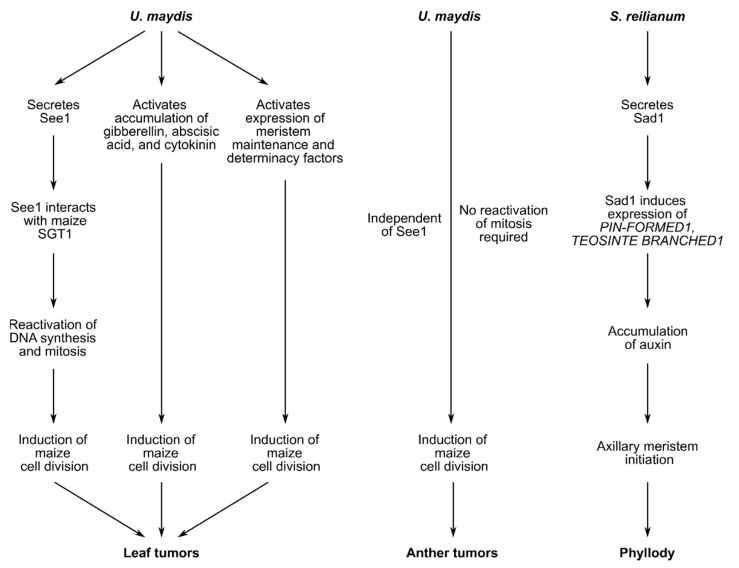
Summary of signals involved in leaf tumor formation, anther tumor formation or phyllody. Tumor formation in maize leaves infected with *U. maydis* depends on secretion of See1, accumulation of phytohormones, and expression of meristem factors. Together these factors reactivate cell division in mitotic inactive leaf tissue. Anther tumor formation is independent of the effector See1 and does not require reactivation of mitosis. Instead, mitotically active cells undergo additional rounds of periclinal and anticlinal division. The *S. reilianum* effector Sad1 induces expression of maize genes and subsequently accumulation of auxin leading to axillary meristem formation.

In leaves, *U. maydis*—induced tumors consist of hypertrophic mesophyll cells and hyperplastic bundle sheath cells. Leaf tumor formation is influenced by the U. maydis effector See1, which is translocated into the plant cell and interacts with maize SGT1 (SUPPRESSOR OF G2 ALLELE OF *SKP1*), a cell cycle and immune regulator (Figure 3*)*. In detail, See1 inhibits MAPK-triggered phosphorylation of maize SGT1 at a monocot-specific phosphorylation site. This leads to reactivation of maize DNA synthesis and mitosis needed for tumor formation [12]. See1 can also be found in other smut fungi. Domain swap experiments revealed that the BRAC motif, which is mainly represented in proteins involved in cell cycle checkpoints and in response to DNA damage, might be partially responsible for the tumor formation phenotype [62]. It was recently speculated that mutation of the *U. hordei* See1 BRAC motif resulted in loss of function. This idea is supported by the fact that the BRAC domain is conserved in the tumor-forming smut M. pennsylvanicum [48,62]. On the other hand, the BRAC sequence is also conserved in *S. reilianum* and the yeast-like fungi Pseudozyma hubeiensis [62] raising questions about the activity of this effector in non-tumor inducing fungi.

Tumor formation is not completely abolished in leaves infected with *U. maydis* SG200Δ*see1* [12,62]; hence, other factors are required for tumor development. Phytohormones such as gibberellins, abscisic acid, and cytokinin, which control plant growth and development, have been associated with tumor formation (Figure 3). Expression of gibberellin biosynthesis enzymes, as well as gibberellin-responsive genes, is induced during leaf tumor induction [49]. In infected cobs, both abscisic acid and cytokinin levels are elevated [63]. It is worthwhile to mention that application of exogenous cytokinin mimics the tumor-associated reduction in photosynthetic rate, maintenance of nutrient sinks, elevated levels of total soluble sugars, and increased nitrogen accumulation during infection (for review see [64]). In addition to phytohormones, it was recently shown that infection reactivates expression of meristem maintenance and determinacy factors, which might regulate cell division and differentiation in tumors (Figure 3) [65]. Yet, it remains elusive if See1, phytohormones and meristem-regulating factors are acting independently of each other during tumor formation or are (partially) interconnected.

*U. maydis* tumor induction also occurs in reproductive organs. Young maize anthers consist of undifferentiated meristematic layer-1 and layer-2-derived cells. During anther development, layer-1-derived cells differentiate into epidermis cells. A subset of layer-2-derived cells become vasculature and connective tissue. In the theca, a few layer-2-derived cells develop into germline cells, which undergo mitosis prior to meiosis. The remaining layer-2-derived cells undergo two rounds of periclinal divisions to form distinct somatic layers (for review see [66]). During *U. maydis* infection, mitotically active somatic cells in the theca undergo multiple unrestricted rounds of periclinal and anticlinal division without differentiation, resulting in tumors (Figure 3) [67]. In contrast to leaf tumor formation, See1 is not required for tumor development in the highly mitotically active anther tissue [12]. 

*S. reilianum* as well tampers with meristem control and phytohormones to induce changes in floral organ identity, to increase tiller formation, and to trigger suppression of apical dominance in the host plant. This manipulation of the plant is most likely facilitated by the fungal effector Sad1 (suppressor of apical dominance1) (Figure 3). Ectopic expression of Sad1 in *A. thaliana* increases the number of secondary inflorescence branches. This smut effector regulates expression of the maize auxin transporter *PIN-FORMED1* and the maize branching regulator *TEOSINTE BRANCHED1* [68]. In line, auxin levels are increased by 30% in *S. reilianum*—infected inflorescences (Figure 3) [21]. Auxin accumulation, which is essential for axillary meristem initiation in maize, and *S. reilianum* hyphae distribution colocalize during later stages of infection in tassels [69,70]. In vitro, auxin promotes filamentous growth of the fungi. Treatment with the auxin transport inhibitor naphthylphthalamic acid (NPA) rescued floral organ identity, even though the tissue was colonized with fungal hyphae [70]. 

Thus, morphological changes of the plant are dependent on the mitotic activity of host cells and might be strongly associated with the host-cell differentiation program, e.g., phytohormones and meristem-regulating factors. Certainly, sensing of the developmental stage of host cells by the fungus is a prerequisite for induction of morphological changes. This idea is underlined by the fact that *U. maydis* does not form tumors in maize mutants with defects in pre-meiotic anther differentiation and division [71]. Yet how the host cell status is recognized by smuts is unknown.

### 3.4. Signals for Teliospore Formation

Many smuts overwinter as teliospores, which are often formed in the plant´s reproductive tissue. Among the here mentioned smut fungi, *S. reilianum* and *Microbotryum* form teliospores in the flower tissue before fertilization. In contrast, teliospores of *U. hordei* and *T. thlaspeos* replace the plant seeds, resembling closely the original seed morphology. Based on this, one could speculate that different events in the plant life cycle and their spatiotemporal signals are required to induce teliospore development of the different smuts.

For the *S. reilianum*-maize interaction, a close link between fungal development and the plant´s switch from vegetative to reproductive growth has been reported and discussed above [20,70]. This distinct switch in the plant is characterized by the development of the shoot apical meristem into the inflorescence meristem and governed by environmental factors, e.g., daylength and temperatures, but also by endogenous factors, e.g., plant age. The required signal transmission in the plant is based on phytohormones and carbohydrates among others (for review see [72,73,74]). Once the inflorescence meristem is formed, several types of meristems emerge from it. Finally the floral meristem transitions into primordial cells that differentiate into the germline and somatic cells [66]. As mentioned above, many smuts invade undifferentiated cells and follow the sugar distribution hierarchy inside the plant. Accordingly, proliferating hyphae have been found in primordial cells below the meristems, and for *T. thlaspeos,* it has been shown that it invades all newly developing leaves from there [37]. In addition, *S. reilianum* seems to follow the various different meristems inside the maize tassel [70]. This raises the idea that directional growth of smut fungi is dependent on plant meristems, and that their terminal differentiation induces sporulation. Another distinct event in the plant life cycle, which might induce teliospore formation, is fertilization leading to embryo formation and seed filling. Here also, carbohydrate signaling and phytohormones direct plant development and seed filling [75]. In summary, little is known about signals governing where and when to sporulate.

## 4. Summary and Open Questions

**Figure 4:**
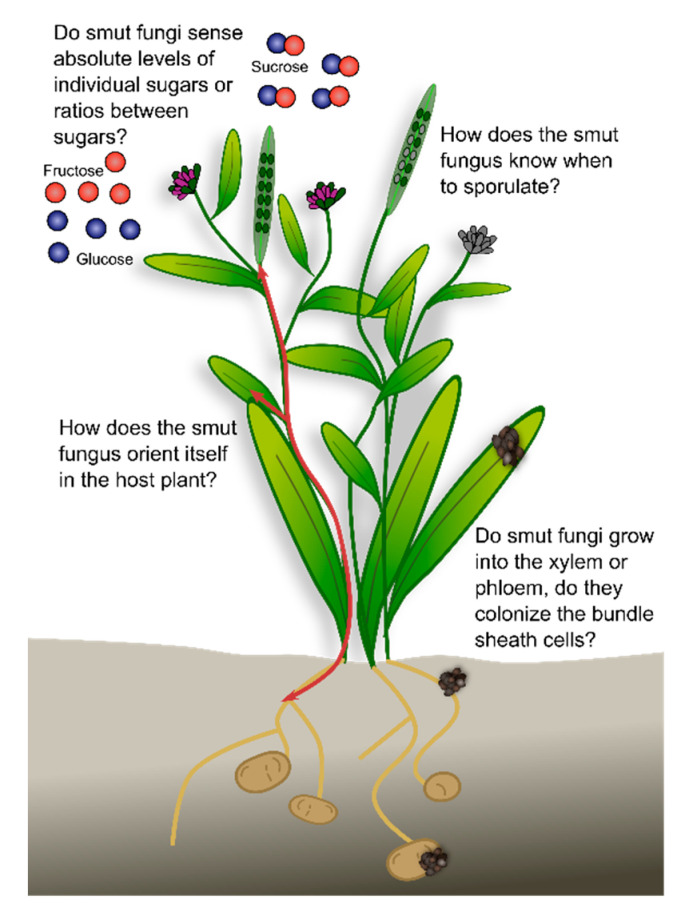
Open questions on smut growth and development inside the plant. For details see text.

As a large group of biotrophic plant pathogens, the smut fungi colonize various host plants with different lifestyles, and accordingly have adapted to their host. Several smuts reproduce in the reproductive organs of the host plants. Prior to sporulation, many smut fungi follow a more or less extended endophytic lifestyle in the host plant without causing major damage. Some species affect plant morphology, while others remain macroscopically undetectable. Some remain in the local tissue while others spread systemically often along the vasculature, a major highway for many plant processes. These differences in infection biology raise several questions (Figure 4):

1. How does the smut fungus orient itself in the host plant? For successful proliferation, it is essential that the fungus perceives plant cues as to what stage the plant is in, where it penetrated the host plant, and where to grow next. Nutrient availability might contribute to this, since it is an easy signal that could have evolved from saprophytes that follow nutrient gradients in their environment.
2. Do smut fungi that follow the vasculature grow into the xylem or phloem, do they colonize the bundle sheath cells, or do they remain in the apoplastic space? Which feeding structure do the different fungi use at the different timepoints during infection? For many species, the existing microscopic data do not allow answering this question. Advanced imaging techniques combined with genetically modified reporter strains and plant lines will enable time-resolved high-resolution tracking of the fungal infection process.
3. Do smut fungi sense absolute levels of individual sugars or ratios between sugars? Sugars are important nutrients for both the plant and the fungus. In addition, modification of the sucrose: glucose ratio activates the plant immune system (SWEET immunity). By sensing absolute sugar levels, the fungus would be able to follow sugar gradients to, for example, reach the vasculature or the reproductive organs. By sensing sugar ratios, the fungus might be able to tame its virulence during the biotrophic phase to avoid activation of the plant-immune system.
4. How does the smut fungus know when to sporulate? Following spatiotemporal orientation, the fungus needs to decide when to sporulate. In annual plants, a general sporulation signal might be the initiation of the reproductive organs, or fertilization. In perennial host plants, the fungus needs to decide which parts of the mycelium should sporulate. Similar to the case of annual plants, this could be induced by a flowering signal that is absent from the vegetative tissues.

Even though smut fungi act parasitic on a variety of plants, including annual and perennial hosts, certain patterns of infection, growth, and sporulation seem to partially overlap. Based on this, one might speculate that the mostly unknown signals, derived from the host and the pathogen, that govern spatiotemporal orientation and development of the fungi, are shared. Recent advancements in transformation technologies (smuts and their hosts), microscopic imaging, and sequencing will help to study the similarities and differences in the group of smut fungi in the near future. This will greatly enhance our understanding of biotrophic plant pathogens and, by this, might also contribute to securing crop yield.
